# 3D-Printed Pacifier-Shaped Mouthpiece for fMRI-Compatible Gustometers

**DOI:** 10.1523/ENEURO.0208-21.2021

**Published:** 2021-10-05

**Authors:** David Munoz Tord, Géraldine Coppin, Eva R. Pool, Christophe Mermoud, Zoltan Pataky, David Sander, Sylvain Delplanque

**Affiliations:** 1 Swiss Center for Affective Sciences, University of Geneva, 1202 Geneva, Switzerland; 2 Department of Psychology, University of Geneva, 1205 Geneva, Switzerland; 3 Department of Psychology, UniDistance Suisse, 3900 Brig, Switzerland; 4 Department of Medicine, University of Geneva, 1205 Geneva, Switzerland

**Keywords:** 3D printing, flavor, gustometer, mouthpiece, fMRI, taste

## Abstract

Gustometers have made it possible to deliver liquids in functional magnetic resonance imaging (fMRI) settings for decades, and mouthpieces are a critical part of these taste delivery systems. Here, we propose an innovative 3D-printed fMRI mouthpiece inspired by children’s pacifiers, allowing human participants to swallow while lying down in an MRI scanner. We used a large sample to validate the effectiveness of our method. The results suggest that the mouthpiece can be used to deliver taste stimuli by showing significant clusters of activation in the insular and piriform cortex, which are regions that have been consistently identified in taste processing. This mouthpiece fulfills several criteria guaranteeing a gustatory stimulus of quality, making the delivery precise and reliable. Moreover, this new pacifier-shaped design is simple and cheap to manufacture, hygienic, comfortable to keep in the mouth, and flexible to use in diverse cases. We hope that this new method will promote and facilitate the study of taste and flavor perception in the context of reward processing in affective neuroscience, and thus, help provide an integrative approach to the study of the emotional nature of rewards.

## Significance Statement

The neuronal networks underlying taste perception have been of great interest in the investigation of fundamental processes, as well as the investigation of the mechanisms involved in a variety of eating disorders. However, the study of food rewards requires specific equipment, combining both precision and comfort. Here, we provide a design for a customizable, fMRI-compatible mouthpiece capable of delivering different liquids in a precise and consistent manner to participants while lying down in a scanner. Additionally, this new pacifier shaped design is comfortable in the mouth and allows for the correction of imaging artifacts when combined with appropriate methods. This open-source design can be used to customize and manufacture mouthpieces to meet unique demands of specific research projects and individual needs.

## Introduction

Studying the neuronal pathways of chemical senses (i.e., olfaction and gustation) requires special equipment. It is relatively easy to make olfactometers (Coppin, 2020), and the same statement may be even truer for gustometers (Canna et al., 2019). The gustometer is a tool specifically designed to deliver liquids. Some gustometers have been used for almost 20 years (O’Doherty et al., 2002; Small et al., 2003). However, while mouthpieces, which are a critical part of the gustatory delivery system (Andersen et al., 2019; Canna et al., 2019), have not been updated in that time, the number of publications on the topic has kept increasing over the years.

**Figure 1.**
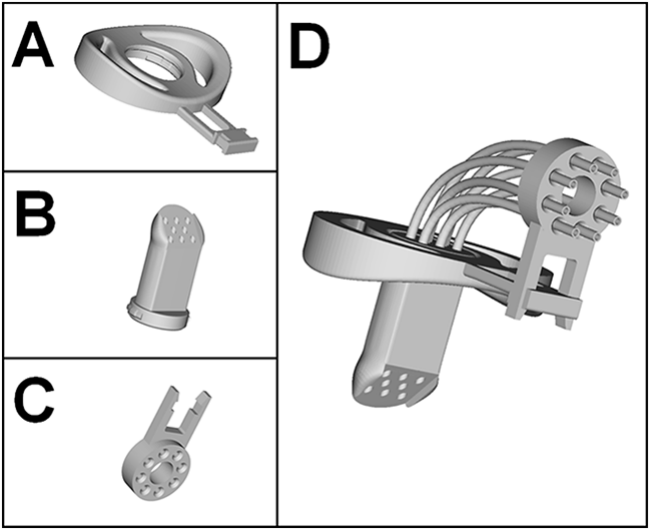
3D representation of the fMRI-compatible mouthpiece. Detailed 3D representation of (***A***) the mouth shield, (***B***) the mouthpiece, (***C***) the tube guide, and (***D***) the complete mouthpiece assembled with eight tubes.

Here, we propose an innovative 3D-printed functional magnetic resonance imaging (fMRI)-compatible mouthpiece, which fulfills several criteria for a quality gustatory stimulus. First, this new mouthpiece (Fig. 1) allows participants to swallow liquids while lying down in a scanner, with their heads immobilized in a given position, and can remain comfortably in the mouth for a considerable amount of time without requiring any particular effort. Indeed, this design, inspired by children’s pacifiers, replaces biting sticks which are sometimes used, onto which participants need to apply pressure with their teeth. Moreover, with biting sticks, it is sometimes necessary to take into account individual dental impressions (Goto et al., 2015). Second, up to eight different liquids can be delivered with this mouthpiece in a precise and consistent manner, making it possible to minimize somatosensory variations and allowing researchers to target the same taste buds over each repetition.

### Mouthpiece description

The mouthpiece, inspired by children’s pacifiers, consists of three parts: a mouth shield, an elongated teat, and a tube guide. These three pieces are separately printed using natural polylactic acid (PLA), a biodegradable plastic made from corn. Other plastics can be used, but it remains the researcher’s responsibility to comply with the health standards of the country in which they are conducting experiments with this mouthpiece.

An oval mouth shield (Fig. 1*A*), with a curvature adapted to the morphology of the face, holds the mouthpiece comfortably on the lips. A cylindrical teat (40 mm long × 22 mm in diameter) is inserted and clipped in the center of the mouth shield. This teat receives the tubes at one extremity and directs the liquids to the tongue at the other extremity (Fig. 1*B*). The part of the teat that goes into the mouth and is intended to come into contact with the tongue is beveled on one side and rounded on the other. This allows for an easy contact of the tongue with the teat to deliver drops of liquid comfortably and accurately. Depending on the research needs, up to eight tubes with an external diameter of 2.5 mm (±0.3 mm) can be inserted into the teat. The last piece is a tube guide (Fig. 1*C*) that is clipped onto the mouth shield and allows the tubes to be at a 90° angle so that they run along the body of the participant lying on the MRI bed (Fig. 1*D*). The 3D printing files (stl) that we supply at https://github.com/munoztd0/Mouthpiecegusto include seven versions with a diameter of 2.5 ± 0.3 mm in steps of 0.1 mm. All these versions make it possible to choose the parts that best fit together depending on the 2.5-mm tubes the researchers use and allows them to adjust the mouthpiece for different types of liquid or viscosity levels.

Since our plans are freely available, the mouthpiece can be made by any laboratory with access to a 3D printer, or it could otherwise be made by any 3D printing service company. It can be manufactured in quantity for a very low price (0.5 USD of material per piece). This makes it intrinsically hygienic, since each participant can get an individual mouthpiece. Moreover, the printing material can easily be adapted to comply with different countries’ sanitary regulations. Our mouthpieces were made out of natural PLA, which is safe when used in contact with food (Conn et al., 1995). Finally, the mouthpiece does not require any modification to any preexisting apparatus and will seamlessly fit most gustometer setups.

## Materials and Methods

### Participants

This study was part of a larger experiment related to a different study question (NCT03347890) in which 97 right-handed participants were recruited. The experiment took place from 2018 to 2020 (i.e., before the COVID-19 pandemic). The study was approved by the Swissmedic ethical committee. All participants gave written informed consent and received 100 Swiss francs (the equivalent of 100 USD$) for their participation in one session. In total, 12 participants were excluded from the analysis because of missing or incomplete data (five MRI and seven behavioral). We report data on the 85 remaining participants (55 female; mean age, 37.3 ± 12.4; min–max, 18–67 years). No predetermined sample size was estimated via statistical methods. All participants reported a normal sense of smell. All participants were asked to fast overnight because of the experiment occurring in the morning.

### Stimuli preparations

Milkshake preparations were made from a mix of milk (300 g) and ice cream (60 g) for a total of 71 kcal/100 g. Potassium chloride (KCl, 1.8 g) and sodium bicarbonate (NaHCO_3_, 0.21 g) were diluted in 1 l of distilled water to recreate an artificial tasteless saliva solution. This main solution was then used to create less concentrated versions to be able to match each individual’s perception of a tasteless solution. In total, there were four different tasteless concentrations (1/1, 3/4, 1/2, and 1/4) and three flavors of milkshake (strawberry, chocolate, and vanilla). We chose an individually adjusted tasteless solution as the control stimulus instead of plain water because water has been shown to have an inherent taste (Bartoshuk et al., 1964). The two solutions were taken out of the fridge simultaneously (30 min before the experiment) and delivered at ambient temperature. We took each participant’s preferred milkshake flavor and the saliva solution that tasted the most neutral to them (i.e., closest to 50 on a scale from 0 to 100) as the two conditions for the experiment.

### Gustometer

Single channel syringe pumps (Chemyx OEM) were used to achieve high flow control. Two syringes of up to 60 ml were connected via 8-m-long food grade polyurethane tubing (external diameter = 4 mm, inner diameter = 2.5 mm) to a 1-m-long food grade polytetrafluoroethylene (PTFE) tubing (external diameter = 2.5 mm, inner diameter = 1.9 mm) and to the mouthpiece at a delivery rate of 1 ml/s. The syringe pumps were connected to a 16-port RS-232 rackmount device server (Moxa, Nport 5610) and then controlled via transmission control protocol (TCP) using specific C libraries designed for stimulus presentation software (MATLAB or python). Although it is out of the scope of this article, readers can refer to Andersen et al. (2019), Canna et al. (2019), or Iannilli et al. (2015) for detailed instructions on how to set up an fMRI-compatible gustometer.

### Taste reactivity task

**Figure 2.**
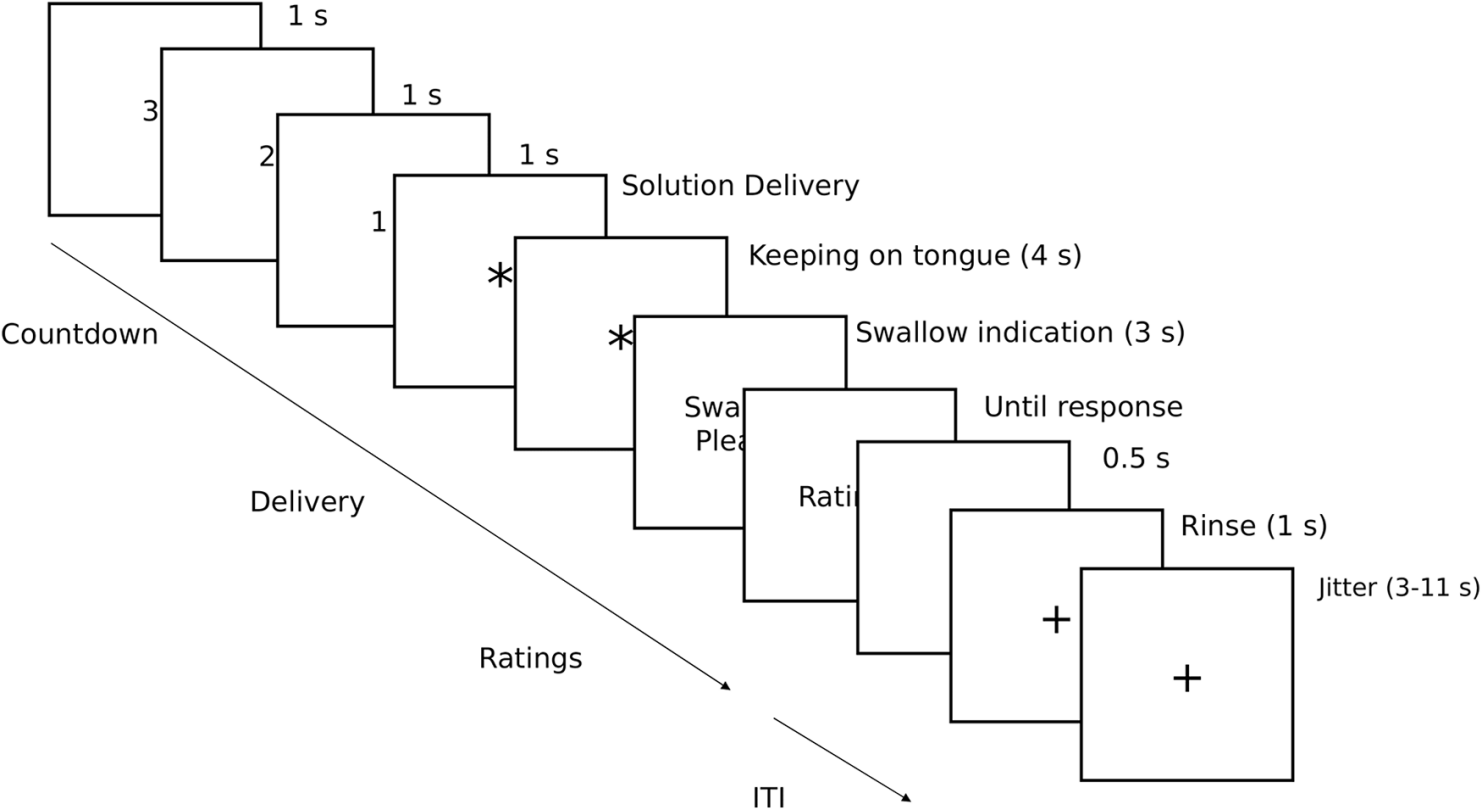
Task procedure. The sequence of the taste reactivity task, administered while participants were lying in the scanner. After a brief countdown, the participants were shown a fixation cross followed by an asterisk cueing the delivery of either a milkshake or a tasteless solution. They were asked to keep the solution on their tongue for 4 s and then prompted to swallow it. During this period, they were asked their perceived taste pleasantness and intensity of the solution. The experimental trials were intertwined with rinse trials to cleanse their palates.

A taste reactivity task was administered while participants were lying in the scanner. The task consisted in the evaluation of the perceived pleasantness and intensity of two different stimuli: a milkshake and a tasteless solution. During each trial, 1 ml of the solution was administered, and the delivery order of the two conditions was randomized within each participant. Participants were visually guided through the task with on-screen indications. First, they saw a 3-s countdown before the solution delivery, followed by an asterisk indicating to keep the solution on their tongue until they saw the swallow indication “swallow please” (Fig. 2). We asked them to wait 4 s before swallowing to avoid adding movement noise to the blood oxygen level-dependent (BOLD) response. Since they were lying down, the mouthpiece was placed in a such a way that the solution was delivered at the center of the participant’s tongue, and we expected that the solution would slide down to the back of their tongue in the 4-s gustation period. The experimental trials were intertwined with rinse trials to cleanse the participants’ palates with 1 ml of water. All 40 evaluations (20 per solution) were done on visual analog scales displayed on a computer screen. Participants had to answer through a button-box placed in their hand. The visual scales for the intensity report ranged from “not perceived” to “extremely intense”; and from “extremely unpleasant” to “extremely pleasant” for liking ratings.

### Data acquisition

The collection of the responses was controlled by a computer running MATLAB (version R2015a; MathWorks). The presentation of the stimuli was implemented using Psychtoolbox (version 3.0; Brainard, 1997). The acquisition of the neuroimaging data were performed via a 3-Tesla MRI system (Magnetom Tim Trio, Siemens Medical Solutions) supplied with a 32-channel head coil following a gradient echo (GRE) sequence to record BOLD signal. We recorded forty echoplanar imaging (EPI) slices per scan with an isotropic voxel size of 3 mm. Our scanner parameters were set at: echo time (TE) = 20 ms, repetition time (TR) = 2000 ms, field of view (FOV) = 210 × 210 × 144 mm, matrix size = 70 × 70 voxels, flip angle = 85°, 0.6-mm gap between slices. Structural whole brain T1-weighted (T1*_w_*) images (isotropic voxel size = 1.0 mm) were acquired, as well as dual gradient *B*_0_ field maps (Fmaps) for each participant to correct for inhomogeneity distortions in the static-field.

### Preprocessing

We combined the fMRI of the Brain (FMRIB) Software Library (FSL, version 4.1; Jenkinson et al., 2012) with the Advanced Normalization Tools (ANTS, version 2.1; Avants et al., 2011) to create a pipeline optimized for the preprocessing of our neuroimaging data.

A challenge of fMRI gustometry is that the BOLD signal is highly prone to movement artifacts, and thus, the swallowing of liquid solutions while lying down produces significant deglutition artifacts. To offset this loss of signal-to-noise ratio (SNR), we followed Griffanti et al. (2017) a rigorous protocol based on an fMRI independent component analysis (ICA) to remove artifacts.

We used the multivariate exploratory linear optimized decomposition tool (MELODIC; Beckmann and Smith, 2004) to decompose our raw BOLD signal into independent components (ICs). The ICA-based strategy for motion artifact removal has been shown to be more reliable to remove motion-induced signal variations than regressions from motion parameters (Pruim et al., 2015). Two researchers independently hand classified a sample of 20 participants’ IC into two categories: clear artifact (e.g., motion/deglutition, susceptibility, or blood flow in arteries) or potential signal. The categorizations were then compared between the two judges, and each discrepancy was discussed until an agreement was reached (inter-rater reliability = 93%). The manually classified components obtained by this process were used to train a classifier using a random forest machine learning algorithm (Breiman, 2001). Leave-one-out testing, where we iteratively left one participant out of the training data and tested the classifier accuracy on the left-out participant, at the optimal sensitivity (threshold = 5) resulted in a median 94% true positive rate (i.e., the percentage of true signal accurately classified). Consequently, we applied the FMRIB’s ICA-based X-noiseifier (FIX) to automatize the denoising of our BOLD signal (Salimi-Khorshidi et al., 2014).

Field maps were then applied to correct geometric distortions and ANTS was used for a diffeomorphic co-registration of the preprocessed functional and structural images in the Montreal Neurologic Institute (MNI) space, using the nearest-neighbor interpolation and leaving the functional images in their native resolution. Finally, we applied a spatial smoothing of 8-mm full width half maximum (FWHM).

### Data analysis

Statistical analyses of the behavioral data were performed with R (version 4.0; R Core Team, 2019). We report Cohen’s *d_z_* and the 95% confidence intervals (CIs) as estimates of effect sizes for the paired *t* tests (Lakens, 2013), as well as a Bayes factor (*BF*_10_) quantifying the likelihood of the data under the alternative hypothesis relative to the null hypothesis (Morey et al., 2015).

The Statistical Parametric Mapping software (SPM; version 12; Penny et al., 2011) was used to perform a random-effects univariate analysis on the voxels of the image time series following a two-stage approach to partition model residuals to take into account within-participant and between-participant variance (Holmes and Friston, 1988; Mumford and Poldrack, 2007).

We specified a subject-level general linear model (GLM) for each participant and added a high-pass filter cutoff of 1/128 Hz to eliminate possible low-frequency confounds (Talmi et al., 2008). Each regressor of interest was derived from the onsets and duration of the stimuli and was convoluted with a canonical hemodynamic response function (HRF) into the GLM to obtain weighted parameter estimates (*β*). The subject-level GLM consisted of six regressors: (1) the trial, (2) the reception of the milkshake solution, (3) the reception of the tasteless solution, (4) water rinsing, (5) the question about solution pleasantness, and (6) intensity. No motion correction regressor was included in the GLM, since we already removed motion components from the signal. Group-level statistical *t* maps were then created by combining subject-level estimated β weights (milkshake > tasteless) and residuals.

A multiple comparisons correction was done using the Analysis of Functional NeuroImages software (AFNI; version 20.2; Cox, 1996). First, we used the *3dFWHMx* function with the spatial auto-correlation flag on to estimate the intrinsic spatial smoothness of the noise in our data. The estimate values were averaged across participants and then used in the corrected *3dClustSim* function (Cox et al., 2017) to determine, via Monte Carlo simulation of the noise field, a minimum cluster extent corrected for multiple comparisons (at *α* = 0.05). This guarantees that a group of individual voxels under an uncorrected height threshold of *p *<* *0.001 with a greater cluster size than the minimum extent would only occur <5% of the time.

We report the minimum extent threshold, the cluster’s peak MNI coordinates, and the number of consecutive significant voxels at *p *<* *0.001 within the cluster (*k*). Finally, we display the statistical *t* maps of our group results for the milkshake > tasteless contrast surviving cluster-level correction overlaid on a 3D semi-inflated surface brain template in the MNI space.

### Code and data accessibility

The computer code used to produce the mouthpiece as well as to preprocess and analyze the data are available in a publicly hosted software repository (https://github.com/munoztd0/Mouthpiecegusto). Unthresholded statistical *t* maps are available on the Neurovault platform (https://neurovault.org/images/442236/).

## Results

**Figure 3.**
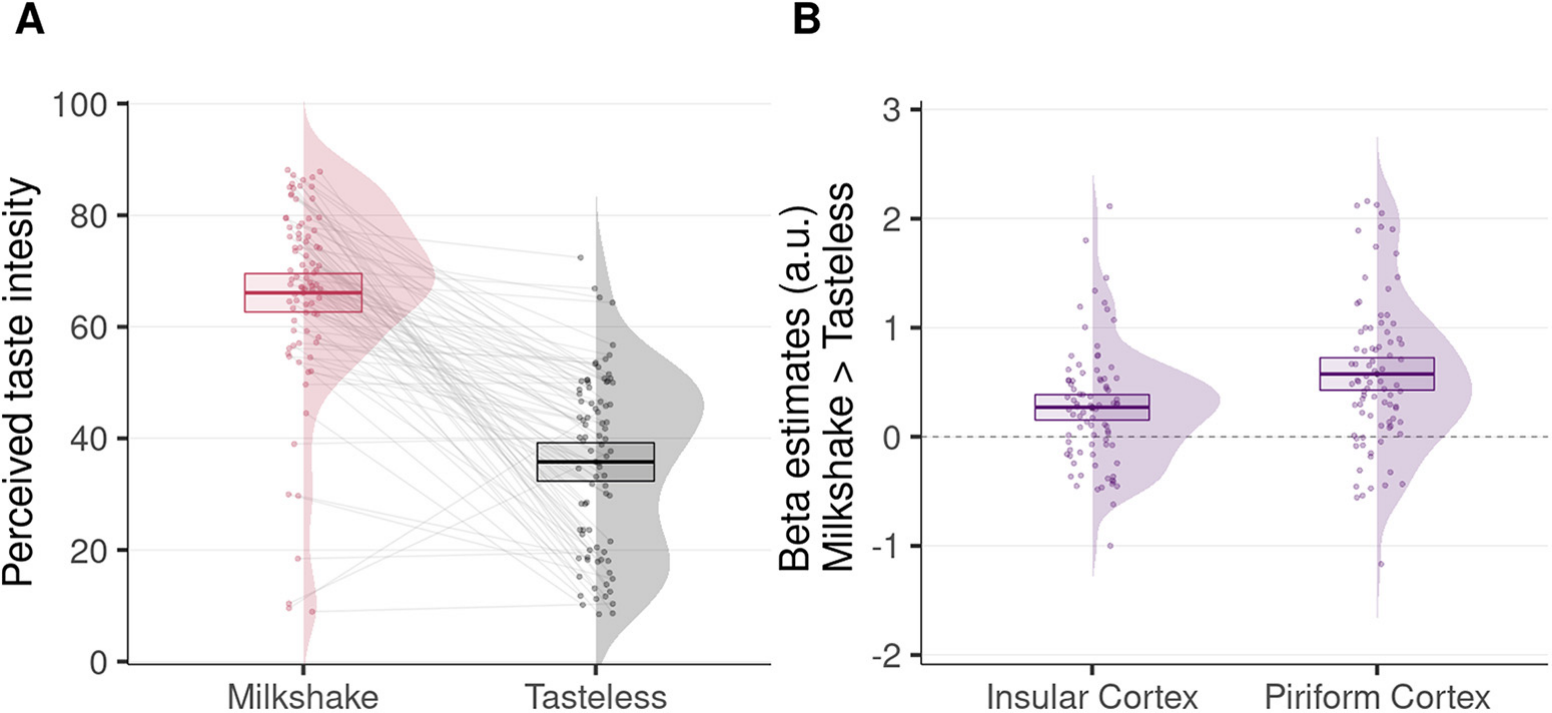
Behavioral and fMRI results. ***A***, Individual estimates, densities, and overall mean of perceived taste intensity of the milkshake and the tasteless solutions. ***B***, Individual β estimates, densities, and overall means of the milkshake > tasteless contrast across participants during taste delivery extracted from voxel clusters within the insular and piriform cortex. Error bars represent 95% CI (*n* = 85).

We analyzed the taste intensity ratings using a paired *t* test to compare the two conditions (milkshake or tasteless). As expected, participants rated the milkshake solution as significantly more intense than the tasteless solution ( $\mu =30.33,\;SE\pm 2.5,\;t_{\left(84\right)}=12.40$, *p *<* *0.001, *d_z_* = 1.35, 95% CI $=[1.04,1.63],\;BF_{10}=9.23\times 10^{20}$; see Fig. 3*A*).

**Figure 4.**
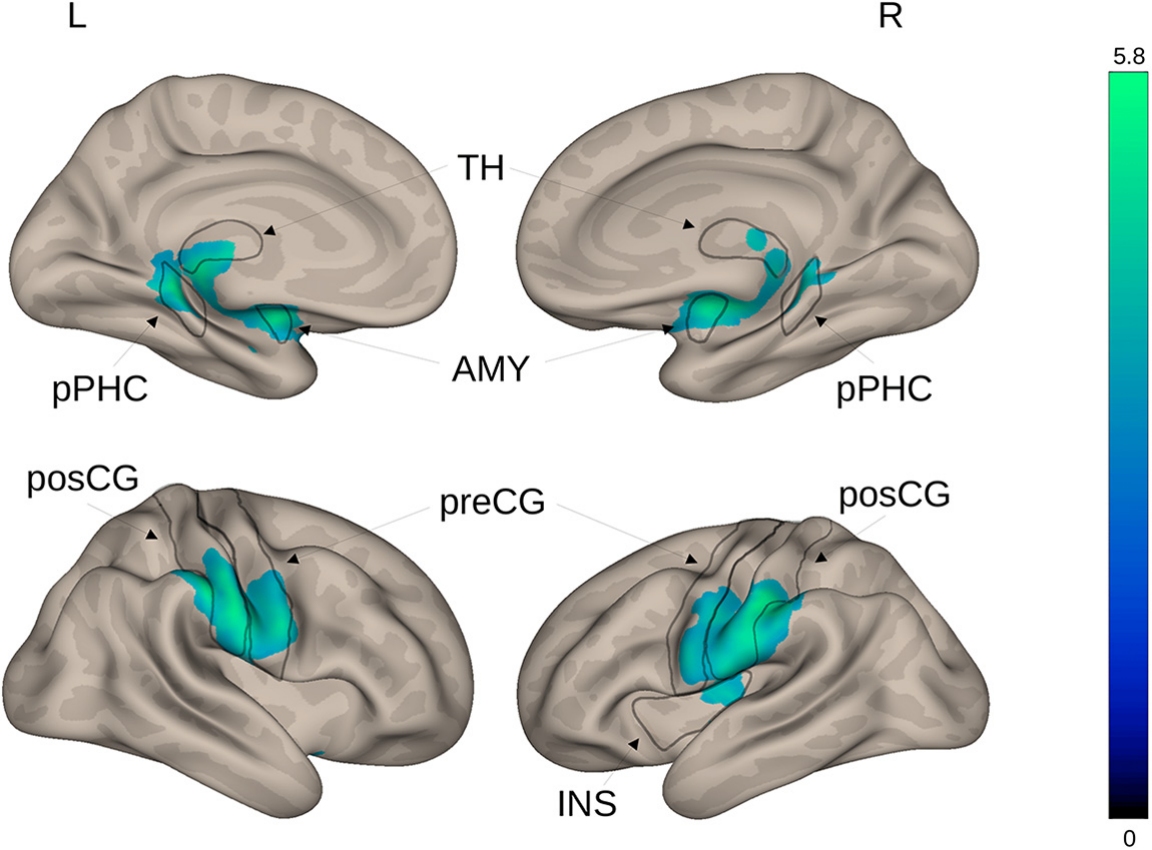
Neural correlates of taste. Regions in which the BOLD signal positively correlates with the magnitude of the contrast milkshake > tasteless (*n* = 85). Statistical *t* maps are shown with a threshold of *p *<* *0.001 and a minimum cluster extent threshold (corrected for multiple comparisons) of 123 voxels. Color scale bar shows *t* statistic values. AMY, amygdala; pPHC, posterior parahippocampal cortex; TH, thalamus; posCG, postcentral gyrus; preCG, precentral gyrus; INS, insula. Detailed results are presented in Extended Data Figures 4-1, 4-2.

We report the results from our group-level analysis using a height threshold of *p *<* *0.001, with a minimum cluster extent threshold corrected for multiple comparisons at *p *<* *0.05 (*k *=* *123 voxels). For the taste reactivity task, the pleasant solution (milkshake > tasteless) activated the primary olfactory (piriform) cortex bilaterally (right: MNI [*xyz*] = [−22 −3 −14], *k *=* *282; left: MNI [*xyz*] = [21 −6 −14], *k *=* *149), the primary gustatory (middle insular) cortex (left: MNI [*xyz*] = [21 −6 −14], *k *=* *149), and the primary somatosensory (postcentral/parietal operculum) cortex (Figs. 3*A*, 4).

10.1523/ENEURO.0208-21.2021.f4-1 Extended Data Figure 4-1 Summary Results of BOLD Activations during the Taste Reactivity Test. Download Figure 4-1, PDF file.

10.1523/ENEURO.0208-21.2021.f4-2 Extended Data Figure 4-2 **Observed power.(A)** One would need 53 participants to reproduce our results within the insular cortex with a power of 90% and an α = 0.05. **(B)** One would need 29 participants to reproduce our results within the piriform cortex. Download Figure 4-2, PDF file.

To compute observed power calculations within our two regions of interest, namely the insular and piriform cortex, we extracted the averaged β values from within these regions and calculated their standardized effect size (*d_z_*). We report a *d_z_* = 0.41 for the insula and *d_z_* = 0.56 for the piriform cortex and estimate that to reproduce these results at 90% power and *α* = 0.05, then 53 or 29 participants are needed for the insula and the piriform cortex, respectively (details of the power analysis, including power curves, are provided in the Extended Data Fig. 4-2).

## Discussion

In this article, we presented a 3D-printed fMRI-compatible mouthpiece for the study of human taste and flavor perception in MRI settings. After describing this mouthpiece, we reported the results of a 3 Tesla fMRI study providing evidence that this mouthpiece allows us to obtain an effective measure of brain related activity during the consumption of gustatory stimuli.

In this large sample (*n* = 85) study, we demonstrate the effectiveness and validity of our procedure by showing significant clusters of activation within the same regions that have been reported throughout different meta-analyses on taste (Yeung et al., 2017) and olfaction (Seubert et al., 2013). More precisely, we found strong activations of the following: (1) the left middle insular cortex, which has consistently been identified as the human primary gustatory cortex (Buck and Bargmann, 2000; Small and Faurion, 2015), (2) the postcentral/parietal operculum gyrus, which has been reported to be the primary cortex for oral somatosensory representation in humans (Boling et al., 2002), and (3) the anterior medial temporal lobes, including the hippocampal formation and the amygdaloid complex, that have also both been found to play a crucial role in food intake (Davidson et al., 2009; Petrovich, 2011; Coppin, 2016). Our results are also in agreement with an asymmetric model of taste perception (Iannilli and Gudziol, 2019), where right-handed populations tend to have stronger left dominance in the insula.

Importantly, we encountered some limitations that should be addressed. First, some participants reported that a 40-mm-long mouthpiece was a bit too long and thus, uncomfortable. This can easily be alleviated by printing a shorter mouthpiece in those cases. We also tried to extend our setup to a non-MRI context, where participants would be seated in an upright position. It appeared that the liquids did not flow as consistently and precisely as they did in a lying position, and suggests that the prototype would have to be modified for such contexts. In a few cases and during intensive use, we also noticed that the plastic could become porous, so that the joints between the tubes and the teat were no longer perfectly sealed. As a result, some participants reported that the rinsing liquid had run down their cheeks. However, this did not prevent the stimuli from being sent, but it is something that the researchers should monitor. One option might be to choose a less porous plastic that is still within the country’s legislative constraints on plastics permitted for food contact. Moreover, we think it is important to tell participants to place their tongue in such a way as to let the solutions flow without blocking the teat to deliver drops of liquid comfortably and accurately.

Another caveat to the interpretation of our results is that we have not controlled for temperature and mechanosensory information. Existing devices have managed to control for this by having the delivery tubes running through a water bath at a controlled temperature (e.g., 37°C) as well as delivering a continuous spray of the solution over the tongue via a spray head to avoid mechanical simulation effects (Iannilli et al., 2015; Andersen et al., 2019). While a spray taste delivery system has proved its efficacy and reliability for event related taste pulses, it could not be used with our test stimuli (i.e., milkshake) because of its high viscosity. This feature was important for our design since we wanted to be able to use realistic feeding paradigms (e.g., milkshakes) as it has been recently advocated by the new “good practice in food-related neuroimaging” (Smeets et al., 2019). We think however that the solutions from Iannilli et al. (2015) and Andersen et al. (2019) could be compatible with our mouthpiece. We suggest future investigators that either want to study or to control for the effects of temperature and mechanosensory information on taste perception to take these methods into account.

Additionally, we unfortunately could not provide a direct comparison between the data collected with our new design and data collected from other commonly used mouthpieces. This will hopefully be possible in future investigations through an ever-increasing number of data sharing initiatives.

To conclude, the main advantages of this mouthpiece are its low cost, flexibility, ease to produce and fMRI-compatible design. Any lab with access to a 3D printer can make one or could otherwise get them made by any 3D printing service company since our plans are freely available. But most importantly, it is flexible and can be modified for any particular case. It can easily comply with different countries’ sanitary regulations or be adjusted for different types of liquid or viscosity levels. It also does not require any modification to any preexisting apparatus and will integrate to most gustometer setups without any additional work.

More theoretically, affective neuroscience could benefit from the inclusion of more studies in olfaction and taste using primary rewards. This could provide the means for an integrative approach to study the emotional nature of reward (Nummenmaa and Sander, 2020). We think that this new method could help promote the use of primary rewards (e.g., milkshakes) instead of more widely used food pictures to measure hedonic processes. This is extremely important because, not only does it allow a direct comparison to be drawn with the animal literature on innate food rewards, but it also helps avoid reward type-dependent neural circuits of secondary rewards (Sescousse et al., 2013; Nakamura et al., 2020). Moreover, taste consumption can induce an affective experience in itself rather than a representation of the affective experience (i.e., pictures of food), which is a crucial property to properly study reward processing (Pool et al., 2016).

## Synthesis

Reviewing Editor: Ifat Levy, Yale University School of Medicine Decisions are customarily a result of the Reviewing Editor and the peer reviewers coming together and discussing their recommendations until a consensus is reached. When revisions are invited, a fact-based synthesis statement explaining their decision and outlining what is needed to prepare a revision will be listed below. The following reviewer(s) agreed to reveal their identity: Emilia Iannilli, Grace Shearrer.

In this work, the authors propose a new mouthpiece for a gustatory delivery system typically used in taste paradigms to study the gustatory cortex. The design is inspired by children’s pacifiers and can be connected to a maximum of 8 separate tubes for a total of 8 different solutions. The mouthpiece is disposable and realized in polylactic acid (PLA) with a 3D printer for a meager price per piece. The plastic material of realization makes it feasible for use in an MRI scanner. The authors report an fMRI experiment using the mouthpiece, in which they show BOLD response in areas traditionally associated with taste.

Improved taste administration is needed, the idea of creating a mouthpiece with a 3D printer is innovative, and the paper is well written. However, the authors’ statements comparing the new device to other techniques do not seem to be substantiated. A direct comparison to existing technology is critical for this paper to advance the field. How is this mouthpiece better than ’traditional’ gustometer mouth pieces? Below we provide some detailed comments.

- The idea for this mouthpiece is innovative, but it is more simplistic compared to other solutions. The complex organization of taste buds and thermo- and mechanosensory receptors on the tongue makes it difficult to achieve a pure gustatory stimulation. To mask unwanted sensory stimulation, such as temperature or mechanosensory, others have proposed solutions based on a constant spray of liquid at a controlled temperature, to induce habituation. This approach is presented in Iannilli et al. (2015, JofNeurosciMethods - A gustatory stimulation), where the mouthpiece is specially designed for an event-related experiment, also used by Andersen et al. (2019, BehResearch Meth . A new gustometer: Template). Another example is the specific intraoral device designed by Goto et al. (2014, JofNeurosciMethods -High resolution time-intensity recording with synchronized solution delivery system for the human dynamic taste perception), which is able to deliver the taste stimulus to a precise location of the tongue. These other techniques and the differences from the method proposed here should be discussed.

- Why is the mouthpiece designed to target the center of the tongue? We know that taste papillae are highly dense on the tongue surface at the very back, on the edge, or in the anterior two-thirds of the tongue (see: Nature Vol 444, 2006- J. Chandrashekar et al. The receptors and cells for mammalian taste). Since the mouthpiece is disposable, couldn’t it be designed based on the size of each subject’s tongue, to target, for example, the anterior two-thirds of the tongue?

- Motion and participant discomfort are the predominant sources of error in taste administration. Is there a way to quantify how much better this mouthpiece performs compared to a traditional mouthpiece? Perhaps evaluating motion (framewise displacement, or mm of motion from a mean position such as the metrics produced in fsl’s MCFLIRT)?

- The manuscript makes a point to highlight the sanitary nature of this mouthpiece. Is this because the authors suggest printing a unique mouthpiece for each subject? Are there any other factors that make this mouthpiece more sanitary? Compared to what and based on what measures?

- The abstract states “this mouthpiece fulfills several criteria guarantying a gustatory stimulus of quality, making the delivery more precise and reliable.” It is unclear what the current mouthpiece is being compared to.

- How was the stimulus temperature controlled? Please expand.

- How did the authors verify the normal olfactory function of the participants?

- Was the taste reactivity task visually or auditory guided? How did the subject know to keep the solution on its tongue for 4s? Please explain.

- What motion correction (if any) was included in the GLM model?

- The authors mention that the new 3dClustSim is used, however there are two “new” methods. Is this the “Newer” or “Newest” (using terminology from https://afni.nimh.nih.gov/pub/dist/doc/program_help/3dClustSim.html)?

- The primary gustatory response has been observed in both left and right insula (please see the review Iannilli & Gudziol, 2018, Brain Research, Gustatory pathway in humans: A review of models of taste perception and their potential lateralization). Can the authors discuss their result of left lateralized activation in the context of the literature?

minor points:

- Line 68 the money unit is missing (200 ?)

- Figure 4: Please report the unit of the color-coded bar.

- Rewrite sentence at line 89: “This two solution where then used as the main two stimuli for the rest of the experiment"

- Line 103 ‚A’ instead of ‚An’

- Make sure to spell out the full term the first time an acronym is used.

## Author Response

Dear Dr. Ifat Levy and reviewers,

We would like to thank the reviewers and you for your constructive feedback about our manuscript “3D printed pacifier-shaped mouthpiece for fMRI-compatible gustometers", and for giving us the opportunity to improve it further in this revised version. We have carefully addressed the points that the reviewers have raised and revised our manuscript accordingly (new in bold and old in strikethrough).

We thank you and the reviewers for your time and consideration.

With our best regards,

On behalf of all authors

Reviewer’s comments

1 - Improved taste administration is needed, the idea of creating a mouthpiece with a 3D printer is innovative, and the paper is well written. However, the authors’ statements comparing the new device to other techniques do not seem to be substantiated. A direct comparison to existing technology is critical for this paper to advance the field. How is this mouthpiece better than ’traditional’ gustometer mouthpieces?

We would like to thank you for the positive feedback concerning our article. We would also like to thank the reviewer for his/her important point: although we are very enthusiastic about this new device and believe it is worth making it available to the scientific community, we did not compare it to other devices. We consequently rephrased the whole paper to avoid any misleading claim. We hope our new phrasing will convey its novelty without suggesting direct comparisons to other devices. More specifically, we changed:

"This mouthpiece fulfills several criteria guarantying a gustatory stimulus of quality, making the delivery more precise and reliable.” Abstract.

"Additionally, this new pacifier shaped design is comfortable in mouth and allows for imaging artifacts correction when combined with appropriate methods. This new pacifier-shaped design also helps reduce mouth discomfort and potential imaging artifacts.” Significant statement.

"Here, we propose an innovative 3D printed functional magnetic resonance imaging (MRI) compatible mouthpiece which fulfills several criteria for guarantying a gustatory stimulus of quality.” Lines 12-14.

2 - The idea for this mouthpiece is innovative, but it is more simplistic compared to other solutions. The complex organization of taste buds and thermo- and mechanosensory receptors on the tongue makes it difficult to achieve a pure gustatory stimulation. To mask unwanted sensory stimulation, such as temperature or mechanosensory, others have proposed solutions based on a constant spray of liquid at a controlled temperature, to induce habituation. This approach is presented in Iannilli et al. (2015, JofNeurosciMethods - A gustatory stimulation), where the mouthpiece is specially designed for an event-related experiment, also used by Andersen et al. (2019, BehResearch Meth. A new gustometer: Template). Another example is the specific intraoral device designed by Goto et al. (2015, JofNeurosciMethods -High resolution time-intensity recording with synchronized solution delivery system for the human dynamic taste perception), which is able to deliver the taste stimulus to a precise location of the tongue. These other techniques and the differences from the method proposed here should be discussed.

The point raised by the reviewer reveals that we may not have emphasized enough an essential aspect of gustometers: there are different mouthpieces that allow to address different research questions. The mouthpiece we are proposing is obviously not designed to answer all these questions. It has strengths and weaknesses, and we hope that the new version of the manuscript will present them in a more objective way.

Part 1: Temperature and mechanosensory information

While the solution used in Iannilli et al. (2015) and Andersen et al. (2019) proved its efficacy and usefulness it could not be used with our test stimuli (milkshake) because of the high viscosity. As pointed in Iannilli et al. (2015): “The gustometer can use all liquids having a viscosity in the range of water.” (Iannilli et al., 2015, p. 15).

We thus tried to provide a new solution towards more realistic feeding paradigms (e.g. milkshakes) which has been advocated by the new “good practice in food-related neuroimaging” (Smeets et al., 2019) and is increasingly been used in reward processing and eating disorder research (e.g Gobbi et al., 2020; Shearrer et al., 2018; Sadler et al., 2021).

Moreover, Andersen et al. (2019) mention custom-made mouthpieces which there is, to our knowledge, no template design available before now:

"The pumps deliver liquids through plastic tubing and can be connected to commercially available or custom-made mouthpiece.” (Andersen et al., 2019, p. 2732)

We thus aimed to provide such template for new investigators to build on.

We agree temperature and mechanosensory information are important to control for, and now state so in our discussion.

"Additionally, a caveat to the interpretation of our results is that we have not controlled for temperature and mechanosensory in- formation. Existing devices have managed to control for this by having the delivery tubes running through a water bath at a con- trolled temperature (e.g. 37{degree sign}) as well as to deliver a continuous spray of the solution over the tongue via a spray head to avoid mechanical simulation effects (Iannilli et al., 2015; Andersen et al., 2019). While a spray taste delivery system has proved its efficacy and reliability for event related taste pulses it could not be used with our test stimuli (i.e. milkshake) due to its high viscosity. This feature was important for our design since we wanted it to be able to use more realistic feeding paradigms (e.g. milkshakes) as it has been recently advocated by the new “good practice in food-related neuroimaging” (Smeets et al., 2019). We think however that the solutions from Iannilli et al. (2015) and Andersen et al. (2019) could be compatible with our mouthpiece. We suggest future investigators that either want to study or to control for the effect of temperature and mechanosensory information on taste perception to take these methods into account.” Lines 272-290.

Part 2: Intra-oral devices

While Goto et al. (2015) intra-oral devices are probably able to deliver the taste stimulus in a more precise manner, our design removes the need for the participant to apply pressure with their teeth which could lead to jaw fatigue/ discomfort and provoke large artifacts from teeth clenching (Grundlehner and Mihajlovic, 2018). It also removes the need to have a dentist take dental impressions of each participant which can time, can be uncomfortable and is cost consuming.

We now address this in our manuscript:

"Indeed this design-inspired by children’s pacifiers-replace ‘biting sticks’ biting sticks’ sometimes used, onto which participants apply pressure on with their teeth and to sometimes have to take into account individual dental impressions (e.g. Goto et al., 2015).” Lines 18-21.

3 - Why is the mouthpiece designed to target the center of the tongue? We know that taste papillae are highly dense on the tongue surface at the very back, on the edge, or in the anterior two-thirds of the tongue (see: Nature Vol 444, 2006- J. Chandrashekar et al. The receptors and cells for mammalian taste). Since the mouthpiece is disposable, couldn’t it be designed based on the size of each subject’s tongue, to target, for example, the anterior two-thirds of the tongue?

We designed the mouthpiece to deliver rather than target the solution at the center of the tongue. This choice was motivated by the fact that the participants were lying down. We consequently expected that the solution would slide down from the center to the back of their tongue by the 4 s gustation period. Albeit targeting the anterior two-thirds of the tongue sounds like a great idea for sitting positions, we did not choose to do so here because we were afraid it would have been harder to reliably target this location since the movement amplitude of the tongue gets larger towards the anterior part of the tongue.

To clarify this point, we added to the manuscript:

"The mouthpiece was placed such as to deliver the solution at the center of the participant’s tongue, this was because they were lying down, and we expected that the solution would slide down to the back of their tongue by the 4 s gustation period.” Lines 120-124.

Of course, one of the main advantages of this design is its flexibility. The availability of the source 3D files makes it easy to modify it to the size of each participant’s tongue and/or target different locations of the tongue.

4 - Motion and participant discomfort are the predominant sources of error in taste administration. Is there a way to quantify how much better this mouthpiece performs compared to a traditional mouthpiece? Perhaps evaluating motion (framewise displacement, or mm of motion from a mean position such as the metrics produced in FSL’s MCFLIRT)?

We unfortunately cannot test this since we have not acquired other traditional mouthpiece data to compare it to.

However, we were eager to provide some information on this point to the reviewer and report here our framewise displacement data. To achieve that we pulled publicly available raw de-identified data from the MRIQC Web-API (mriqc.nimh.nih.gov). This project host more than 200’000 individual time- series which we filtered from to get normative data from similar acquisition parameters (TR < 1.9 and < 3.0 s; TE > 0.015 and < 0.05 s). This left us with 834 data points to compare with.

Although this method did not allow to compare the influence of our mouthpiece to another one, it is interesting to note that when looking at the average framewise displacement (FD) most of our distribution lies above 0.2 mm which is considered as the FD threshold. More strikingly, we see that the number of timepoints above the FD threshold is very high (median = 0.38, Q1 = 0.29, Q3 = 0.51) and above 100 for every participant. These two metrics strongly suggest, as rightly pointed out by the reviewer, that motion artifacts are an important issue to tackle when administering taste solutions.

Additionally, we compared our raw BOLD time-series average FD and the number of timepoints above the FD threshold to the same time-series after ICA-based artifact removal. This comparison shows a clear improvement of the signal since after the artifact removal all the distribution of the average FD lies below the FD threshold.

We are aware this only partly answer the reviewer’s question, but we hope that the ease of availability of this information would allow future investigations on this topic.

Framewise displacement compared to normative data. (A) Average framewise displacement (FD) from each time series compared to 834 crowd-sourced data points with similar scanner parameters from the MRIQC Web-API (mriqc.nimh.nih.gov). The average FD is calculated as the displacement on the surface of a sphere (Power et al., 2012). (B) Number of timepoints above FD threshold compared to the MRIQC Web-API.

Framewise displacement compared before and after preprocessing. (A) Average framewise displacement (FD) from each time series compared to the same time series after artifact removal (see A1 for more information). The average FD is calculated as the displacement on the surface of a sphere (Power et al., 2012). (B) Number of timepoints above FD threshold compared to the same time series after artifact removal.

5 - The manuscript makes a point to highlight the sanitary nature of this mouthpiece. Is this because the authors suggest printing a unique mouthpiece for each subject? Are there any other factors that make this mouthpiece more sanitary? Compared to what and based on what measures?

Yes, the sanitary nature we were mentioning was related to being able to print a unique mouthpiece for each participant and thus not have to repeatedly clean the mouthpiece after each session and possibly deteriorate it. We mention this in the manuscript:

"This makes it intrinsically hygienic since each participant can get an individual mouthpiece.” Lines 29-30.

6 - The abstract states “this mouthpiece fulfills several criteria guarantying a gustatory stimulus of quality, making the delivery more precise and reliable.” It is unclear what the current mouthpiece is being compared to.

As we mentioned in comment 1, we did not test that and thus this sentence was modified in the manuscript:

"This mouthpiece fulfills several criteria guarantying a gustatory stimulus of quality, making the delivery more precise and reliable.” Abstract.

7 - How was the stimulus temperature controlled? Please expand.

We did not control temperature with a thermometer, but the two solutions were taken out of the fridge simultaneously (30 min before the experiment), so we expect that stimuli were both delivered at ambient temperature.

Accordingly, this information was added to the manuscript:

"The two solutions were taken out of the fridge simultaneously (30 min before the experiment) and delivered at ambient temperature.” Lines 86-88.

8 - How did the authors verify the normal olfactory function of the participants?

The olfactory function of all participants was investigated using self-reports. All participants reported a normal sense of smell. Due to the current context, we also want to point out that this assessment as well as the whole study were done before the COVID-19 pandemic.

This information was added to the manuscript:

"This experiment took place from 2018 to 2020, i.e. before the COVID-19 pandemic.” Lines 64-65.

"All participants reported a normal sense of smell.” Line 73.

9 - Was the taste reactivity task visually or auditory guided? How did the subject know to keep the solution on its tongue for 4 s? Please explain.

The taste reactivity task was visually guided. We clarified that it in the manuscript:

"Participants were visually guided through the task with on-screen indications. First, they saw a 3s countdown before the solution was delivery, followed by an asterisk that indicated to keep the solution on their tongue until they saw the swallow indication “swallow please” (see Fig. 2). We asked them to wait 4s before swallowing to avoid adding movement noise to the Blood-Oxygen-Level-Dependent (BOLD) response.” Lines 114-120.

And in the caption of Figure 2:

"The sequence of the taste reactivity test administered while participants were lying in the scanner. After a brief countdown, the participants were showed a fixation cross followed by an asterisk cueing the delivery of either a milkshake or a tasteless solution. They were asked to keep the solution on their tongue for 4s and then indicated to swallow it. At this moment they were asked their perceived pleasantness and intensity of the solution. The experimental trials were intertwined with rinse trials to cleanse their palate.” Fig. 2.

10 - What motion correction (if any) was included in the GLM model ?

We did not include any motion confound regressors in the GLM since we had already removed motion components from the signal. ICA-based strategy for motion artifact removal have been shown to be more reliable to remove motion-induced signal variations (Pruim et al., 2015) than multiple linear regression from motion parameters.

Here is a short summary from Pruim et al. (2015):

"ICA-AROMA and ICA-FIX both yielded improved RSN [resting-state network] reproducibility and decreased loss [...] compared to spike regression and scrubbing. [...] Nuisance regression including realignment parameters was unsuccessful in addressing such effects of secondary motion artifacts. [...] regression strategies minimally reduced the impact of motion. [...] This finding is consistent with previous results (Van Dijk et al., 2012, Power et al., 2012, Satterthwaite et al., 2012, Yan et al., 2013) and further highlights the limited applicability of realignment parameters to model the full complexity of secondary motion-induced variance (e.g. spin history effects).” (Pruim et al., 2015, p. 285)

We added this sentence to the manuscript:

"No motion correction regressor was included in the GLM since we already removed motion components from the signal.” Lines 185-187.

And clarified this in the preprocessing paragraph:

"We used the multivariate exploratory linear optimized decomposition tool (MELODIC; Beckmann and Smith, 2004) to decompose our raw BOLD signal into independent components (IC). ICA-based strategy for motion artifact removal have been shown to be more reliable to remove motion-induced signal variations than regression from motion parameters (Pruim et al., 2015). Two researchers independently hand classified a sample of 20 participants’ IC into two categories: ’clear artifact’ (e.g., motion/deglutition, susceptibility, or blood flow in arteries) or ’potential signal’. Labels were then compared between the two judges, where each discrepancy was discussed until an agreement was reached (interrater reliability = 93%). Manually classified components obtained by this process were used to train a classifier using random forest machine learning algorithm (Breiman, 2001). Leave-one-out testing-where we iteratively left one participant out of the training and tested the classifier accuracy on the left-out participant-at the optimal sensitivity (threshold = 5) resulted in a median 94% true positive rate (i.e., the percentage of ’true signal’ accurately classified). Consequently, we applied the FMRIB’s ICA-based X-noiseifier (FIX) to automatize the denoising of our BOLD signal Salimi-Khorshidi et al. (2014). “ Lines 158-178.

11 - The authors mention that the new 3dClustSim is used, however there are two “new” methods. Is this the “Newer” or “Newest” (using terminology from AFNI ?)

Thank you for pointing this out, the short answer is that we used the “newer” method. However, when we talked about the “new” 3dClustSim function it was not related to the “obsolete” vs. “newer” vs. “newest” methods for estimating the spatial smoothness but to differentiate the “corrected” 3dClustSim function from the “buggy” one from before 2015.

Here is a short summary from Cox et al. (2017) about the incident:

"There was a bug in 3dClustSim. [...] The bug, pointed out in an e-mail, was a flaw in how 3dClustSim rescaled the simulated 3D noise grid after smoothing to bring the variance of the values back to 1.0 (for ease of later p value thresholding). This rescaling was off due to improper allowance for edge effects (effectively, zeros “outside” the grid were included in the smoothing), with the result being that the cluster-size thresholds computed were slightly too small, so that the FPR would end up somewhat inflated. During part of the work leading to (Eklund et al., 2015, 2016), this bug was fixed in May 2015 and noted in the regular and publicly available log of AFNI software changes.” (Cox et al., 2017, pp. 153-154).

We clarified this in the manuscript:

"The multiple comparisons correction was done using the Analysis of Functional NeuroImages software (AFNI; version 20.2; Cox, 1996). First, we used the 3dFWHMx function with the spatial auto-correlation flag on to estimate the intrinsic spatial smoothness of the noise in our data. The averaged value over participants of these estimates were then given in the corrected 3dClustSim function (Cox et al., 2017) to determinate-via Monte Carlo simulation of the noise field-given an uncorrected height threshold of p < 0.001, a cluster of a size of 123 voxels or greater would occur less than 5% of the time.” Lines 190-199.

12 - The primary gustatory response has been observed in both left and right insula (please see the review Iannilli & Gudziol, 2019, Brain Research, Gustatory pathway in humans: A review of models of taste perception and their potential lateralization). Can the authors discuss their result of left lateralized activation in the context of the literature?

Thank you for bringing this important paper to our knowledge. We integrated it to our discussion:

"Our results are also in agreement with an asymmetric model of taste perception (Iannilli and Gudziol, 2019) where right-handed population tend to have stronger left dominance in the insula.” Lines 251-254.

12 - Line 68 the money unit is missing (200 ?).

The money unit was redacted for double-blind review about our location. But we can say that the amount is similar to 100 USD$ for one session.

We modified this part of the manuscript :

"All participants gave written informed consent and received 100 200 [4. redacted for double-blind review] (the equivalent of 100 USD$) for their participation to one the whole session.” Lines 66-69.

13 - Figure 4: Please report the unit of the color- coded bar.

We added this to Figure 4:

"Color scale bar shows t-statistic values.” Fig. 4.

14 - Rewrite sentence at line 89: “This two solution where then used as the main two stimuli for the rest of the experiment”.

Thank you for pointing out these errors, we corrected them:

"We took each participant’s preferred flavor of milkshake and the saliva solution they found the closest to neutral (i.e., closest to 50) as the two conditions for the experiment, namely milkshake and tasteless. This two solution where then used as the main two stimuli for the rest of the experiment.” Lines 86-91.

15 - Line 103 “A” instead of “An"

This has been modified:

"An taste reactivity test ...” Line 109.

16 - Make sure to spell out the full term the first time an acronym is used.

Accordingly, these terms have been modified:

functional magnetic resonance imaging (fMRI)

polytetrafluoroethylene (PTFE) tubing

controlled via transmission control protocol (TCP)

Blood-Oxygen-Level-Dependent (BOLD)

Functional Magnetic Resonance Imaging of the Brain (FMRIB)

Analysis of Functional NeuroImages (AFNI)

References

Andersen, C. A., Alfine, L., Ohla, K., and H¨ochenberger, R. (2019). A new gustometer: Template for the construction of a portable and modular stimulator for taste and lingual touch. Behavior Research Methods, 51(6):2733-2747.

Cox, R. W. (1996). Afni: software for analysis and visualization of functional magnetic resonance neuroimages. Computers and Biomedical Research, 29(3):162-173.

Cox, R. W., Chen, G., Glen, D. R., Reynolds, R. C., and Taylor, P. A. (2017). fMRI clustering and false-positive rates. Proceedings of the National Academy of Sciences, 114(17):3370-3371.

Gobbi, S., Weber, S., Graf, G., Hinz, D., Asarian, L., Geary, N., Leeners, B., Hare, T., and Tobler, P. (2020). Reduced neural satiety responses in women affected by obesity. Neuroscience, 447:94-112.

Goto, T. K., Yeung, A. W. K., Suen, J. L. K., Fong, B. S. K., and Ninomiya, Y. (2015). High resolution time-intensity recording with synchronized solution delivery system for the human dynamic taste perception. Journal of Neuroscience Methods, 245:147-155.

Grundlehner, B. and Mihajlovic, V. (2018). Handling noise and artifacts. In Narayan, R., editor, Encyclopedia of Biomedical Engineering, volume 3, pages 223-239. Elsevier-Hill.

Iannilli, E., Beger, M., Fu¨rer, R., and Hummel, T. (2015). A gustatory stimulator. Journal of Neuroscience Methods, 255:12-16.

Iannilli, E. and Gudziol, V. (2019). Gustatory pathway in humans: A review of models of taste perception and their potential lateralization. Journal of Neuroscience Research, 97(3):230-240.

Pruim, R. H., Mennes, M., Buitelaar, J. K., and Beckmann, C. F. (2015). Evaluation of ICA-AROMA and alternative strategies for motion arti- fact removal in resting state fMRI. NeuroImage, 112:278-287.

Sadler, J. R., Shearrer, G. E., Papantoni, A., Yokum, S. T., Stice, E., and Burger, K. S. (2021). Correlates of neural adaptation to food cues and taste: the role of obesity risk factors. Social Cognitive and Affective Neuroscience.

Shearrer, G. E., Stice, E., and Burger, K. S. (2018). Adolescents at high risk of obesity show greater striatal response to increased sugar content in milkshakes. The American Journal of Clinical Nutrition, 107(6):859- 866.

Smeets, P. A., Dagher, A., Hare, T. A., Kullmann, S., van der Laan, L. N., Poldrack, R. A., Preissl, H., Small, D., Stice, E., and Veldhuizen, M. G. (2019). Good practice in food-related neuroimaging. The American Journal of Clinical Nutrition, 109(3):491-503.
