# Erratum: Munoz Tord et al., “3D-Printed Pacifier-Shaped Mouthpiece for fMRI-Compatible Gustometers”

**DOI:** 10.1523/ENEURO.0219-23.2023

**Published:** 2023-07-07

In the article “3D-Printed Pacifier-Shaped Mouthpiece for fMRI-Compatible Gustometers,” by David Munoz Tord, Géraldine Coppin, Eva R. Pool, Christophe Mermoud, Zoltan Pataky, David Sander, and Sylvain Delplanque, which published online on September 22, 2021, one author’s funding was not listed. Géraldine Coppin was supported by an Eccellenza Grant from the Swiss National Science Foundation (Grant 181094). The full funding statement appears below and the online version has been corrected.

This work was supported by the Firmenich SA Research Grant UN9046. G.C. was supported by Swiss National Science Foundation Eccellenza Grant 181094.

