# Supplementary material for: 3D-Printed Pacifier-Shaped Mouthpiece for fMRI-Compatible Gustometers
Source: eNeuro. 2021 Oct 5;8(5):ENEURO.0208-21.2021. doi: 10.1523/ENEURO.0208-21.2021 (PMC8496206; doi:10.1523/ENEURO.0208-21.2021)
Supplement: Extended Data Figure 4-1 — Summary Results of BOLD Activations during the Taste Reactivity Test. Download Figure 4-1, PDF file. [file enu-eN-NWR-0208-21-s03.pdf]

Figure 4-1: Summary Results of BOLD Activations during the Taste Reactivity Test

Region	Laterality	Extent	$t(84)$	Coordinates x	y	z
Postcentral gyrus	L	532	5.19	-55	-21	36
Insular cortex	L	532	4.15	-49	-12	11
Postcentral gyrus	R	327	4.96	63	-15	29
Piriform Cortex / Amygdala	R	282	4.78	-22	-3	-14
Parahippocampal cortex	L	282	4.77	-13	-36	-7
Thalamus	L	282	4.33	-12	-30	-1
Piriform cortex / Amygdala	L	149	4.73	21	-6	-14
Parahippocampal cortex	R	149	3.72	15	-30	-7

Note. Thresholding $t(84) > 3.19$, $p < 0.001$, and minimum cluster level simulation extent for multiple comparison correction at $p < 0.05 = 123$. The table shows all local maxima separated by more than 20 mm. Coordinates are expressed in the Montreal Neurological Institute (MNI) space in the left-right, anterior-posterior, and inferior-superior dimensions, respectively.
