# Supplementary material for: 3D-Printed Pacifier-Shaped Mouthpiece for fMRI-Compatible Gustometers
Source: eNeuro. 2021 Oct 5;8(5):ENEURO.0208-21.2021. doi: 10.1523/ENEURO.0208-21.2021 (PMC8496206; doi:10.1523/ENEURO.0208-21.2021)
Supplement: Extended Data Figure 4-2 — Observed power.(A) One would need 53 participants to reproduce our results within the insular cortex with a power of 90% and an α = 0.05. (B) One would need 29 participants to reproduce our results within the piriform cortex. Download Figure 4-2, PDF file. [file enu-eN-NWR-0208-21-s04.pdf]

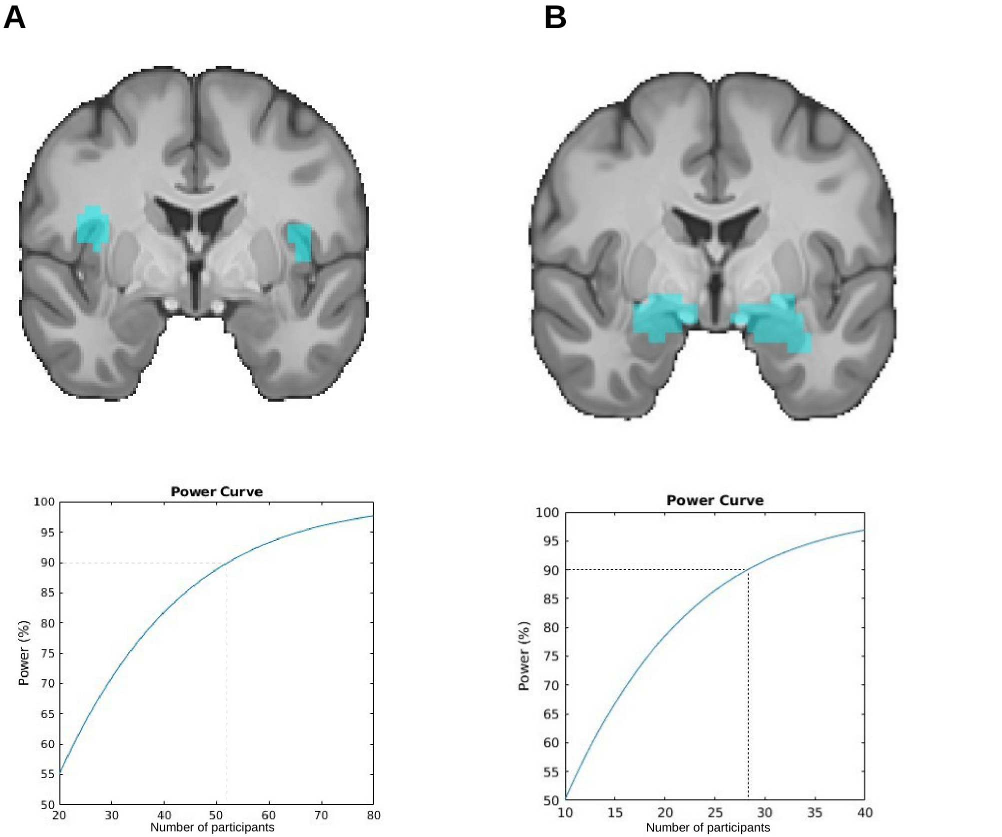

Figure 4-2: **Observed power.** **(A)** One would need 53 participants to reproduce our results within the insular cortex with a power of 90% and an $\alpha = 0.05$. **(B)** One would need 29 participants to reproduce our results within the piriform cortex.
